# Multi-Magnet Cochlear Implant Technology and Magnetic Resonance Imaging: The Safety Issue

**DOI:** 10.3390/audiolres14030034

**Published:** 2024-04-26

**Authors:** Pietro Canzi, Elena Carlotto, Elisabetta Zanoletti, Johan H. M. Frijns, Daniele Borsetto, Antonio Caruso, Luisa Chiapparini, Andrea Ciorba, Giorgio Conte, Nathan Creber, Stefania Criscuolo, Filippo Di Lella, Sebastiano Franchella, Erik F. Hensen, Lorenzo Lauda, Stefano Malpede, Marco Mandalà, Liselotte J. C. Rotteveel, Anna Simoncelli, Anna Chiara Stellato, Diego Zanetti, Marco Benazzo

**Affiliations:** 1 Department of Clinical, Surgical, Diagnostic and Pediatric Sciences, University of Pavia, 27100 Pavia, Italy; pietro.canzi@unipv.it (P.C.); stefanomalpede@gmail.com (S.M.); annachiara.stellato01@universitadipavia.it (A.C.S.); marco.benazzo@unipv.it (M.B.); 2 Department of Otorhinolaryngology, University of Pavia, Fondazione IRCCS Policlinico San Matteo, 27100 Pavia, Italy; 3 Department of Otolaryngology, S. Croce Hospital, 12100 Cuneo, Italy; 4 Section of Otorhinolaryngology-Head and Neck Surgery, Department of Neurosciences, University of Padova, 35127 Padova, Italy; elisabetta.zanoletti@unipd.it (E.Z.);; 5 Department of Otorhinolaryngology, Leiden University Medical Center, 2300 RC Leiden, The Netherlands; j.h.m.frijns@lumc.nl (J.H.M.F.); e.f.hensen@lumc.nl (E.F.H.);; 6 Department of ENT, Addenbrooke’s Hospital, Cambridge University Hospitals NHS Foundation Trust, Cambridge CB2 0QQ, UK; daniele.borsetto1@nhs.net (D.B.); nathan.creber@nhs.net (N.C.); 7 Department of Otology and Skull Base Surgery, Otologic Group, 29121 Piacenza, Italy; antonio.caruso@gruppootologico.it (A.C.); lorenzo.lauda@gruppootologico.it (L.L.); 8 Department of Diagnostic Radiology and Interventional Radiology and Neuroradiology, University of Pavia, Fondazione IRCCS Policlinico San Matteo, 27100 Pavia, Italy; l.chiapparini@smatteo.pv.it (L.C.); s.criscuolo@smatteo.pv.it (S.C.); a.simoncelli@smatteo.pv.it (A.S.); 9 ENT and Audiology Department, University Hospital of Ferrara, 44122 Ferrara, Italy; andrea.ciorba@unife.it; 10 Neuroradiology Department Fondazione IRCCS Ca’ Granda Ospedale Maggiore Policlinico Milan, 20122 Milan, Italy; giorgioconte.unimed@gmail.com; 11 Otolaryngology and Otoneurosurgery Unit, University of Parma, 43126 Parma, Italy; filippo.dilella@unipr.it; 12 Department of Otology and Skull Base Surgery, Azienda Ospedaliera Universitaria Senese, 53100 Siena, Italy; mandal@unisi.it; 13 Audiology Unit, Department of Specialistic Surgical Sciences Fondazione IRCCS Ca’ Granda, Ospedale Maggiore Policlinico Milan, 20122 Milan, Italy; diego.zanetti.bs@gmail.com; 14 Department of Clinical Sciences and Community Health, University of Milan, 20122 Milan, Italy

**Keywords:** cochlear implants, magnetic resonance imaging, safety, Ultra 3D, magnet

## Abstract

Despite the spread of novel-generation cochlear-implant (CI) magnetic systems, access to magnetic resonance imaging (MRI) for CI recipients is still limited due to safety concerns. The aim of this study is to assess and record the experiences of Hires Ultra 3D (Advanced Bionics) recipients who underwent an MRI examination. A multicentric European survey about this topic was conducted focusing on safety issues, and the results were compared with the current literature. We collected a total of 65 MRI scans performed in 9 otologic referral centers for a total of 47 Hires Ultra 3D recipients, including, for the first time, 2 children and 3 teenagers. Preventive measures were represented by scanning time and sedation for children. Head wrapping was used in eight cases, and six of the eight cases received local anesthesia, even if both measures were not needed. Only three patients complained of pain (3/65 examinations, 4.6%) due to the tight head bandage, and one of the three cases required MRI scan interruption. No other adverse events were reported. We believe that these results should encourage MRI execution in accordance with manufacturer recommendations for Ultra 3D recipients.

## 1. Introduction

In recent decades, a growing number of patients have benefited from cochlear implantation for hearing rehabilitation [1]. A cochlear implant (CI) device consists of an external processor and an internal component, which is surgically placed beneath the patient’s skin. The internal component comprises a transmitting coil and a magnet. Historically, these elements have raised concerns regarding MRI safety for CI recipients because of their well-known interaction with the MRI magnetic field. Potential adverse events included demagnetization/polarity reversal, or dislocation of the CI internal part, as well as patient discomfort (e.g., pain or skin swelling) [2]. MRI-related problems may require post-scan intervention, namely medical or surgical management up to explantation. The occurrence of MRI-related adverse events has been documented until recently, which may also be due to poor adherence to MRI safety protocols [3]. Over the years, the number of CI recipients requiring MRI has substantially increased. On the one hand, this is due to the growing number of CI recipients, the broadening indications for CI surgery, and the aging of the population living with CI [4]. On the other hand, the number of MRI scans has been rising because of expanding indication criteria and the availability of MRI services [5,6,7]. In line with this, there is also growth in the pediatric MRI market. This can be explained by the demand for advanced healthcare practiced in the pediatric field as well as its well-known validity as an accurate and radiation-free diagnostic tool [8]. The relentless technological progress has led to a new concept of CI. A particular focus has been placed on internal magnet technology with the aim of minimizing adverse events during MRI scanning and preventive procedures associated with MRI. In 2018, Advanced Bionics AG^®^ (AB-Stäfa, Switzerland) introduced the HiRes™ Ultra 3D^®^ device, with a novel magnet system. The internal unit is equipped with four rotatable diametrically magnetized cylinders placed in a revolving disc, thus allowing for proper alignment with the resonance magnetic field. This device obtained approval for examinations up to 3T without magnet removal or compression bandages [9]. Despite the fact that this technology has been broadly employed for surgical use, in the literature, the experiences documented about the use of MRI in patients with HiRes™ Ultra 3D CI are puzzling. The lack of a standardized system for reporting safety issues and the resulting bias contribute to confusion and anxiety among practitioners. The aim of the present work was to address the issue of MRI scan safety in AB HiRes™ Ultra 3D recipients, including children.

## 2. Materials and Methods

We performed a European multicentric study by distributing a survey to CI centers and collecting information about MRI on AB HiRes™ Ultra 3D CI users. Each center submitted a questionnaire dealing with eventual MRI scanning procedures to patients implanted with an Ultra 3D CI. The investigation was performed after obtaining approval from the institutional review board and medical ethics committee. The questionnaire was inspired by a study by Loth and colleagues [10]. We included 19 items, which were divided into 3 groups (Table 1). The first group included a preliminary question conditioning the inclusion in the study, which asked if the CI recipient had undergone an MRI examination after cochlear implantation. The actual performance of the examination had to be documented with images or at least with a medical report. This was necessary to avoid patient confusion with other radiological procedures and to ensure that the MRI was performed after implantation. The other items of this group concerned patients’ demographic data, such as gender, date of birth, and implantation date. Secondly, details about the MRI examination were investigated. If multiple scans were executed, participants were asked to analyze each MRI scan separately. The timing of the scan in relation to the date of CI placement was required. In addition, technical data about the MRI were collected (scanner manufacturer, magnetic field strength, and maximum specific absorption rate used). Information was also collected on the region of the body scanned and the indication for the MRI. Furthermore, participants were asked if any precautions had been taken before or during scanning. The Section 3 deals with any complications that may have occurred during or after the MRI, i.e., reasons for discontinuation, pain, hearing problems, need for re-fitting, change in fixation of the processor magnet, change in position of the internal device, and any other complaints. If participants said they had pain, they were asked to describe it as mild, moderate, or severe and to indicate its location. The last question was about the need for any special measures or interventions after the MRI. The patients or their parents answered the questions during a visit or by mail, after giving their informed consent. Furthermore, when the provided information was partial, data were collected from implant databases or contacting radiologists. Each center completed the survey for every Ultra 3D recipient who had undergone an MRI scan and provided anonymized data. Safety information represented the overarching goal, and in addition to this, the analysis of possible influencing factors was considered a secondary outcome. The data collected in our study were compared with the existing literature.

## 3. Results

Nine otologic referral centers participated in the survey. A total of 47 patients—19 females and 28 males—met the inclusion criteria and answered the questionnaire. The number of subjects belonging to each center was 7, 1, 12, 5, 5, 2, 11, 1, and 3, respectively. Forty-five participants were adults (mean age at the time of CI 54 years, range 13–81 years), including three teenagers (one was thirteen years old and two were fifteen years old, respectively). Two patients were children; their ages at the time of CI implantation were 12 and 8 months old, respectively. The whole population was implanted unilaterally at the time of the MRI scanning. The results are reported in Table 2 and compared with the current literature.

**Figure 1:**
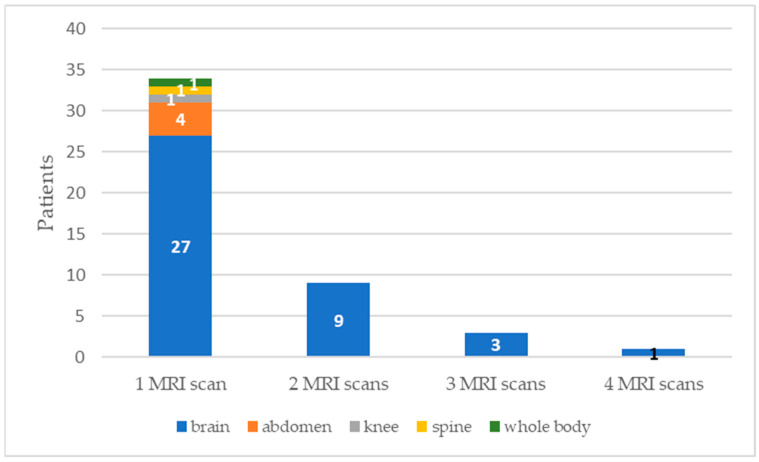
Distribution of patients undergoing single or multiple MRI scans with the examined body region. Each column represents the number of patients who underwent one, two, three, and four MRI scans, respectively. The “1 MRI scan” column is divided with a color code according to the scanned body region. On the other hand, the other columns are uniform because multiple MRI were performed all for the head region.

The mean time interval between implantation and the first MRI scan was 12 months (from 1 to 50). Most of the patients underwent one MRI scan, and thirteen had more than one scan. Three patients received three MRI scans and only one patient underwent four MRI scans. Moreover, 83.1% (54/65) of the exams were performed using a 1.5 T magnetic field, while 16.9% (11/65) were performed with a 3T MRI scanner. The most-scanned body area was the brain, with a total of 89.2% (58/65). Moreover, for the totality of CI recipients submitted to multiple MRIs, the examined region was the brain. Figure 1 shows the distribution of patients undergoing single or multiple MRI scans and their examined body region.

**Figure 2:**
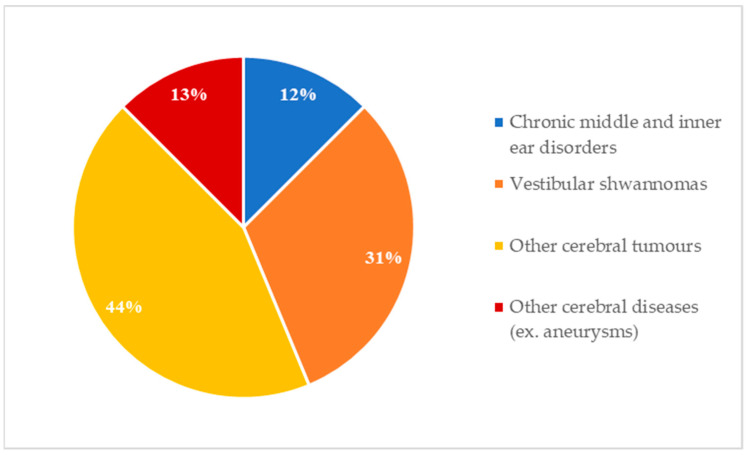
Indication for planned MRI follow-up (32/47 patients): 4 chronic middle and inner ear disorders, 10 vestibular schwannomas, 14 other cerebral tumors, 4 other cerebral diseases (ex. aneurysms).

**Figure 3:**
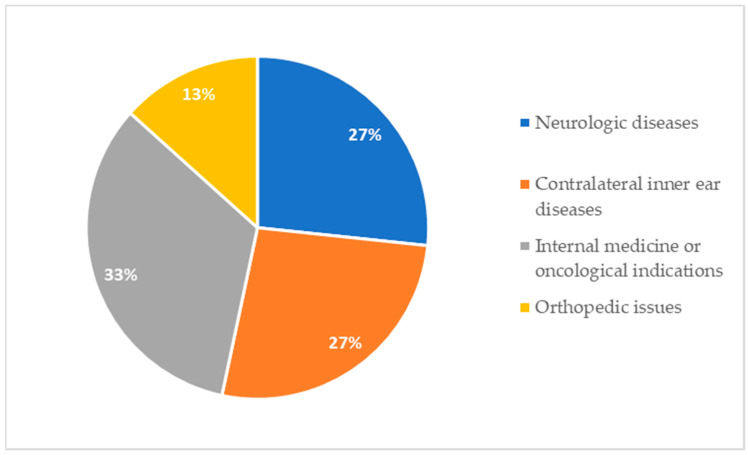
Indication for patients undergoing unplanned MRI scans (15/47 patients): 4 neurologic diseases, 4 contralateral inner ear disease, 5 internal medicine or oncological indications, 2 orthopedic issues.

**Figure 4:**
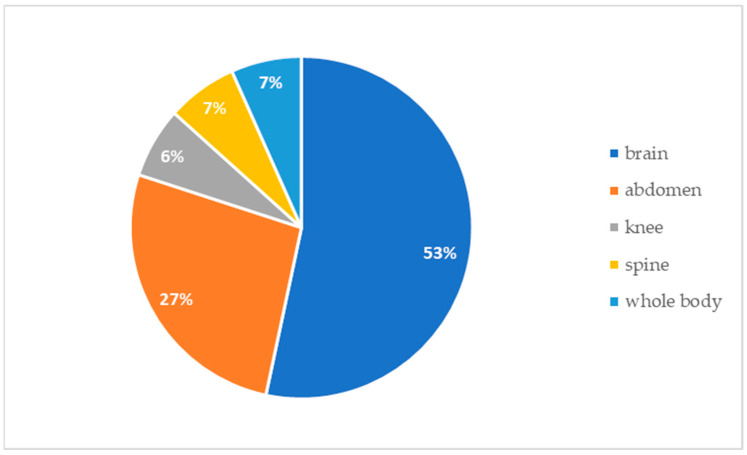
Regions of the body scanned in patients undergoing unplanned MRI scans (15/47): 8 brain, 4 abdomen, 1 knee, 1 spine, 1 whole body.

Concerning indications for MRI scans, in 68.1% (32/47) of patients, the radiologic follow-up was foreseen before implantation (Figure 2). All these scans were of the head. All patients who underwent multiple MRI scans belong to this subgroup. In contrast, 31.9% (15/47) of CI recipients underwent MRI for incoming reasons (Figure 3). Figure 4 shows the body regions scanned in patients who underwent unplanned MRI scans.

**Figure 5:**
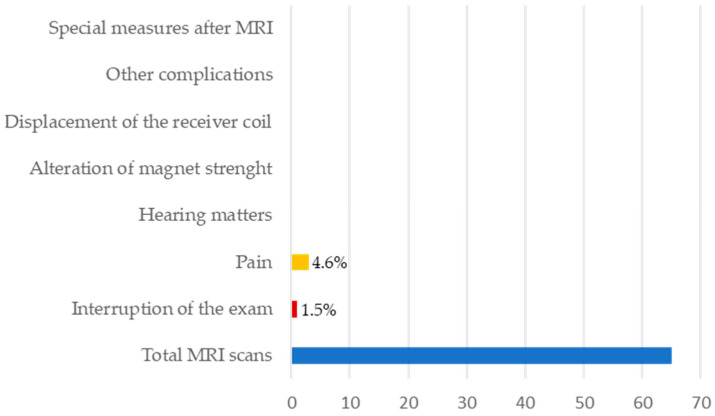
Complications of MRI scans.

**Figure 6:**
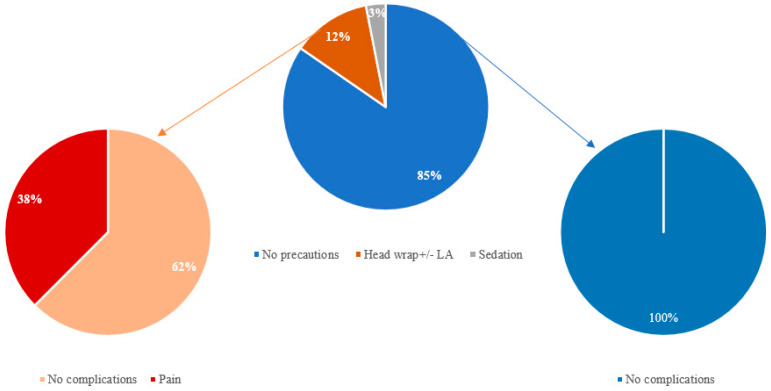
Precautions and relative incidence of complications.

Two centers used preventive measures not required by manufacturer specifications: head wrapping was used for eight patients, and six of the eight patients (belonging to the same center) also received local anesthesia through the infiltration of mepivacaine. The other examinations were performed without constraints, apart from attention to the scanning time, as indicated by the manufacturer, and sedation for children. The scans were almost entirely carried out without complications (Figure 5). Pain over the device bed was the only problem mentioned, reported by three patients (3/65 examinations, 4.6%). The complaint occurred in all three cases in adult patients undergoing a single MRI scan: a brain MRI for headache and seizures, a whole-body MRI for multiple myeloma (interrupted), and an abdominal MRI for a prostate tumor. Pain was classified as mild in two cases and moderate in one, with the latter requiring an interruption of the exam. All the patients reporting pain had head wrapping (see Figure 6). Two of them, including the participant who abandoned the examination, were from the same center and received both a head bandage and anesthetic infiltration around the implant. They both claimed to have experienced pain described as head numbness associated with a tight head bandage. They denied experiencing burning, painful cold, tingling, itching, electrical pain, or pins and needles. The patient who stopped the study because of moderate pain complained of an accompanying burning sensation in the eyes. However, he commented that the bandage was the most uncomfortable experience. In this case, the MRI was not repeated as an 18-fluorodeoxyglucose PET/CT (Positron Emission Tomography/Computed Tomography) was performed to complete the staging of multiple myeloma. The third patient belonged to another center and received head wrapping without anesthesia. He reported feeling mild pain at the site of the bandage, without further details. There were no reports of device functional alterations or hearing matters, even in patients who had complained of pain.

## 4. Discussion

The concern regarding adverse events caused by the interaction between CI and the MRI magnetic field has led to a transversal technological evolution over the years. In 2014, MED-EL^®^ (MED-EL, Innsbruck, Austria) provided an innovative alternative to the traditional axial magnet system by introducing the Synchrony^®^ implant. This CI contains a single rotatable magnet that aligns with the MRI magnetic field. Manufacturer guidelines indicate MRI compatibility up to 3 T without magnet removal or head bandaging [14]. Similarly, Cochlear^®^ (Cochlear Ltd., Sydney, Australia) has commercialized the Nucleus^®^ Profile™ Plus Series with a diametric, self-aligning magnet with no need for a head bandage or magnet removal up to 3 T [15]. In 2018, the innovation launched by AB consisted of magnet equipment composed of four independent magnet bars, and each one is able to rotate on its longest axis and mounted in a rotating frame. The AB HiRes Ultra 3D^®^ multi-magnet system differs from previous single-magnet technologies, allowing for rotation on two axes instead of on one axis. This design allows the magnetization direction to rotate along the magnetic field despite the angle between the CI and the scanner bore, minimizing torque on the implant [16]. Despite technological advances, today, we are still in the “MRI-conditional” era. Namely, each CI manufacturer provides MRI execution restrictions even in the presence of the most advanced models [9,14,15].

Hence, the concern regarding the adverse events due to MRI–CI interaction is not behind us, as demonstrated by the growing interest in the topic [3,10,17,18]. Indeed, a recent article has proposed the implementation of an institutional radiology-administered protocol for patients with different models of CI and auditory brainstem implants requiring MRI scans. This project, which involved an MRI safety team of practitioners, was aimed at designing a streamlined workflow to prevent patients’ discomfort [1]. In general, a limited number of MRI-related complications have been reported in the literature when using new-generation self-aligning magnetic systems [1,3,10]. The safety of the Med-El technology during MRI is well documented in the literature. Bestourous and colleagues, who collected data on MRI-related adverse events in CI users from a Food and Drug Administration (FDA) database, found no reports of matters with the Synchrony CI. [3]. Similarly, the effectiveness of the AB Ultra 3D system in preventing dislocation or demagnetization has been widely demonstrated in preclinical studies [16,19,20]. On the other hand, clinical experiences of MRI scans with this device are still limited. Recently, Johnson and colleagues [1] investigated the occurrence of complications during MRI scans with CIs of different manufacturers and technologies. There were no complications associated with MRI in 22 HiRes™ Ultra 3D recipients. Moreover, three clinical reports underlined the absence of adverse events during MRI scans in patients with HiRes™ Ultra 3D [11,12,13].

To our knowledge, this is the first multicentric European study on the topic aimed at providing a comprehensive overview of otologic referral centers’ experiences. A remarkable aspect of the present study is that it involved both adult and pediatric patients. There is no mention in the literature of MRIs carried out on children with HiRes™ Ultra 3D implants. Here, we describe the experiences of two children (eight and twelve months old, respectively) who underwent MRI under sedation, and three teenagers (one was thirteen years old and two were fifteen years old). Our results confirmed that most MRIs (both 1.5T and 3T) were completed in the absence of adverse events or rigid technical constraints. The exams were conducted according to the manufacturer’s recommendations, which discourage adopting a scanning time of more than 15 min [9]. Moreover, specific absorption rate (SAR) guidelines were respected during the examinations [9]. In addition to this, sedation was performed in younger children. In most of the cases, no pressure bandage or any other physical restraints were used. In 12.3% (8/65) of the cases, belonging to two otologic centers, head wraps were used, even if not required. It is noteworthy that all complications (4.6% of total examinations) occurred in this group of adult CI recipients. There were no further device- or patient-related complications in the whole sample. Among patients who received MRI scans with head bandages, 38% (3/8) complained of tight pressure and dressing-related pain; in one case, the pain resulted in an exam abortion. Given the prevalence of pain in this subgroup and the absence of complaints from non-wrapped patients, we can speculate that the adverse events were due to unnecessary procedures (in particular the bandage), rather than CI–MRI interaction. In accordance with Loth and colleagues, we strongly believe that unrequired measures should be avoided since they can result in patient discomfort and minor adverse events [10]. On the other hand, it is essential to follow the manufacturer’s recommendations, such as scanning time and SAR guidelines. In this regard, the aforementioned FDA database review by Bestourous et al. included a report of magnet dislodgement and pain in a HiRes™ Ultra 3D user. The authors claimed that no head wrap was used on this patient. However, the adoption of necessary scanning precautions was not specified and, in general, poor adherence to the recommended MRI protocols was reported [3].

In addition, in our analysis, we considered the MRI indication and examination target. It must be considered that demands for MRI examination may arise throughout CI recipients’ lives due to incoming diseases, though they may be foreseen before implantation. Likewise, the expansion of CI selection criteria to patients with chronic otitis media and skull base tumors, like vestibular schwannoma (both sporadic and in NF2), has resulted in a large cohort of CI recipients requiring MRI monitoring [21]. Such patients have long been precluded from CI candidacy. Thus, in the present study, for 68.1% (32/47) of the whole cohort, MRI monitoring was indicated as part of their follow-up program. The head was the region scanned in all CI recipients undergoing planned radiological monitoring, most commonly for tumors (Figure 2). Taking into account the incoming indications and the multiple scans, the prevalence of brain MRI in our cohort is 89.2%. Compared to similar studies of CIs with earlier technologies, our brain MRI prevalence is much higher. In fact, in 2017, Grupe and colleagues reported that 49% of cases had the head as the scanned region in their cohort of recipients of implants from different manufacturers [22]. The authors have claimed that MRIs of the head were the most common cause of complications in their sample of patients [22]. In this respect, Eerkens and colleagues have shown that the forces exerted on the CI by MRI depend on the distance between the CI and the bore of the scanner, as well as on the angle between the CI magnet and the magnetic field of the MRI [16]. This aspect is especially relevant for brain MRI, entailing the close proximity of the internal magnet to the scanner bore. However, according to the above-mentioned study by Eerkens et al. [16], torque forces have been shown to be minimal with Ultra 3D magnet technology. Indeed, no major adverse events were reported in our sample irrespective of the examined region and contingent CI position. In addition, in our three cases of pain complaints, there seems to be no correlation with the region examined.

Another topic worth mentioning is the issue of CI-related artifacts in MRI, which has recently been raised as a result of current advances in CI–MRI compatibility. In fact, signal void areas and distortion signals, which occur in the vicinity of the internal unit, cause concerns, in particular for brain MRI. Implant internal units from different manufacturers vary slightly in size. The overall dimensions of the AB Hires™ Ultra 3D are 28.5 mm × 56.2 mm × 4.5 mm, in width, length, and thickness, respectively [23]. The Med-El Synchrony2™ measures 24 mm × 47.3 mm × 4.5 mm [24], while the Cochlear Nucleus Profile™ Plus measures 31 mm × 50.5 mm × 3.9 mm [25]. Despite structural differences, the size of the artifact has been shown to be similar regardless of the device brand and the magnet technology [26]. In the current literature, several strategies have been explored to improve MRI image quality in the presence of the CI magnet, including the adaptation of CI angular position or distance from the external auditory canal [27,28], patient head orientation [29], and MRI algorithm manipulation [30]. In this respect, the application of metal artifact reduction sequences (MARS) has recently been shown to be a valuable additional strategy for improving image quality in the presence of CIs, particularly in the case of skull base disorders requiring MRI monitoring [31,32].

Finally, the current results present some restrictions that deserve to be mentioned. First of all, the majority of our findings are related to a 1.5T magnetic field strength. Secondly, the experience of pediatric patients is limited. Further investigations with a higher number of 3T MRI scansions and a larger proportion of children need to be performed in order to corroborate the existing data.

## 5. Conclusions

We present the first multicentric clinical study about AB HiRes™ Ultra 3D CI adult and pediatric recipients undergoing MRI scanning. The existing data from otologic referral centers seem encouraging. We believe that the evidence from our experience will contribute to overcoming practitioners’ concerns about performing MRI scans on Ultra 3D recipients. In addition to this, we stress the importance of adherence to manufacturer recommendations, thus limiting scanning times and avoiding head bandages.

## Figures and Tables

**Table 1:** Questionnaire submitted to patients/clinicians.

	Questions	Answers
D E M	Have you ever had an MRI scan since you had been provided with an CI? If no, end of questionnaire	
	What is your gender and date of birth?	
	When did you undergo cochlear implantation?	
M R I	When did you undergo your first MRI scan with the CI?	
	Which was the manufacturer of the MRI scanner?	
	Which was the magnetic field strength used?	
	Where conditions regarding maximum specific absorption rate (SAR) observed?	
	Which body region was examined?	
	What was the indication for the execution of the MRI scan?	
	Do you know if and what precautions have been taken regarding MRI?	
C O M P L I C	Has the examination been discontinued? If yes why?	
	Did you have any pain during the examination? If yes, how strong this was? where was it located?	
	Did you experiment any other symptoms during the MRI?	
	Did your hearing get worse after the MRI?	
	Did your need a refit of the speech processor after the MRI?	
	Did you need an external stronger magnet after the MRI?	
	Did the internal magnet change its position during the MRI?	
	Have other complications occurred?	
	Have any special measures been taken after MRI? (Please specify)	

DEM: demographic data; COMPLIC: complications.

**Table 2:** Literature and personal experiences of MRI scans in AB Hires Ultra 3D recipients.

Authors, Year	Subjects	Scans	Scanner Model	Tesla	SAR	Examined Region	Preventive Measures	Complications
Cass et al., 2019 [11]	1	1	Philips	1.5	NA	Brain	NA	None
Fussel et al., 2020 [12]	1	1	NA	1.5	Max 3.2	NA	None	None
Johnson et al., 2023 [1]	22	22	NA	1.5, 3	Max 1	NA	None	None
Canzi et al., 2022 [13]	1	1	Philips	1.5	2.6	Brain	None	None
Our experience	47	65	Philips, General Electric	1.5	Max 3.2 head Max 2.6 body	1 Whole body 1 Spine 1 Knee 4 Abdomen 58 Brain	55 None 8 Wrap (6 with LA) 2 Sedation	62 None 3 Pain (1 exam interruption)
				3	Max 2.6 head Max 2.0 body			

LA local anesthesia, SAR Specific Absorption Rate, and NA not assessed.

## Data Availability

Dataset available on request from the authors.
